# Death Following Vaginal Injection

**Published:** 1874-12

**Authors:** 


### Death following a Vaginal Injection.

The following case is recorded by Lorain as one of those occasional instances where death has apparently been due to some mild manipulatory procedure on the female genitals.

A young girl, sixteen years of age, of delicate constitution, and who had commenced menstruating three months previously, entered the hospital to obtain treatment for vaginitis. A decoction of althæa was employed as an injection at first, but it was soon changed for a solution of nitrate of silver (gr. x.–℥i.). This fluid was injected without force, the greater part returning through the vagina. Immediately afterwards, however, the patient complained of pain in the belly, and it increased so violently that she tossed about in the bed, her eyes became fixed, the skin became cold, the pulse small, and the face pinched. These severe symptoms abated after three hours; but vomiting then set in and continued, though in the following day the pain in the belly had moderated. Some days after, while the vomiting was still persisting, and she was suffering great distress, she gave a sudden cry and died. Tardieu made the autopsy, and found a purulent inflammation of the mucous membrane of the uterus, and the fallopian tubes filled with pus. They were discharging into the peritoneal cavity, and there was diffused peritonitis.—*Allg. Med. Central. Ztg.*, 1, 1874.—*The Record.*

